# Anaerobic coverage as definitive therapy does not affect clinical outcomes in community-onset bacteremic biliary tract infection without anaerobic bacteremia

**DOI:** 10.1186/s12879-018-3184-8

**Published:** 2018-06-15

**Authors:** Pei-Shan Wu, Chien Chuang, Ping-Feng Wu, Yi-Tsung Lin, Fu-Der Wang

**Affiliations:** 1 Department of Medicine, Taipei Veterans General Hospital, Taipei, Taiwan; 2 Division of Infectious Diseases, Department of Medicine, Taipei Veterans General Hospital, No. 201, Sec. 2, Shih-Pai Road, Taipei, 112 Taiwan; 3 School of Medicine, National Yang-Ming University, Taipei, Taiwan; 4 Institute of Emergency and Critical Care Medicine, National Yang-Ming University, Taipei, Taiwan

**Keywords:** Biliary tract infection, Bacteremia, Anaerobic coverage, Antimicrobial stewardship

## Abstract

**Background:**

Antibiotics with anaerobic coverage are widely used for the treatment of biliary tract infection (BTI), even in the absence of isolated anaerobes. The current study aimed to investigate the differences in clinical outcomes in patients with community-onset bacteremic BTIs without anaerobic bacteremia, treated with vs. without anti-anaerobic coverage.

**Methods:**

A retrospective analysis was conducted at a medical center in Taiwan from September 2014 to March 2016. Patients with community-onset bacteremic BTIs without anaerobic bacteremia and who were treated with appropriate antibiotics were analyzed. The clinical outcomes were compared between patients treated with and without anti-anaerobic coverage as definitive therapy after the blood culture reports were available. Multivariable and propensity score-adjusted analysis were used to identify the risk factors associated with treatment failure.

**Results:**

Among the enrolled 87 patients, 63 and 24 patients were treated with and without anaerobic coverage, respectively. *Escherichia coli* (55.2%) and *Klebsiella pneumoniae* (23.0%) were the most common organisms isolated from the blood cultures. The rate of treatment failure (relapse and 28-day mortality) was similar between the groups with and without anaerobic coverage (20.6% vs. 16.7%, *p* = 0.677). Propensity score-adjusted multivariable analysis revealed that definitive therapy without anaerobic coverage was not a predisposing factor for treatment failure (OR = 0.92, 95% CI 0.18–4.67, *p* = 0.916).

**Conclusions:**

Definitive therapy without anaerobic coverage does not affect the outcomes of patients with community-onset bacteremic BTIs without anaerobes isolated from blood. Our results might provide a possible target for antibiotic stewardship interventions in BTIs.

## Background

Biliary tract infection (BTI), including cholangitis and cholecystitis, is a common cause of bacteremia, especially for patients with underlying structural abnormalities, such as choledocholithiasis or malignancy [1]. The causative organisms of BTIs usually originate from the gut flora, including Gram-negative bacteria (70–80%) such as *Escherichia coli* and *Klebsiella* spp. in the majority of cases, followed by Gram-positive bacteria (15–25%) such as *Enterococcus* spp. [2, 3]. Anaerobes are relatively infrequently isolated, with 4–12% from bile cultures and 1–7% from blood cultures. *Bacteroides fragilis* is the most commonly anaerobic pathogen [2–6]. Anaerobes might play a role in BTI patients with a history of biliary surgeries, especially those with a bile duct-bowel anastomosis and common bile duct manipulation [7]. The literature suggests empirical antibiotics with anaerobic coverage in patients with severe disease, biliary-enteric anastomosis, and healthcare-associated BTIs [8]. However, there is a lack of adequate studies investigating whether anaerobic coverage is necessary as the definitive therapy after the culture results are available. Many physicians continue to combine anti-anaerobic agents during the treatment of BTIs even when the cultures yielded no evidence of anaerobic infection.

In terms of bacteremic BTIs, continuing anaerobic coverage after the blood cultures grow only aerobic bacteria is a common practice because of the low culture rate of anaerobes in blood samples. However, unnecessary use of anti-anaerobic agents contributes to the side effect of drug, selective pressure for antibiotic-resistant microorganisms and increased overall treatment costs [9, 10]. In the current era of antimicrobial stewardship [11], it is essential to determine whether anaerobic coverage is necessary in bacteremic BTI patients without anaerobes isolated from blood.

In the present study, we aimed to investigate the differences in clinical outcomes in patients with community-onset bacteremic BTIs treated with and without anaerobic coverage as definitive therapy after the blood culture reports were available.

## Methods

### Study design and patient selection

This retrospective study was conducted at a tertiary medical center, Taipei Veterans General Hospital, in Taiwan. We reviewed the relevant medical and microbiology records to identify consecutive patients older than 20 years of age with community-onset bacteremic BTIs from September 2014 to March 2016. This study was approved by the Institutional Review Board of Taipei Veterans General Hospital.

BTI was diagnosed on the basis of a clinical presentation of fever, right upper quadrant pain, imaging findings (computed tomographic or sonographic) of biliary obstruction, and laboratory findings of hyperbilirubinemia and an elevated serum alkaline phosphatase level [2]. Bacteremic BTI was defined as a compatible clinical syndrome with presence of positive blood cultures consistent with ascending cholangitis or acute cholecystitis [1].

Community-onset bacteremia included community-acquired and healthcare-associated bacteremia [12]. Healthcare-associated bacteremia included patients with a positive blood culture at the time of hospital admission or within 48 h of admission if the patient fulfilled any of the following criteria: (1) hospitalization for 2 days during the preceding 90 days; (2) intravenous therapy at home; (3) chronic dialysis during the preceding 30 days; (4) residing in a nursing home or long-term care facility; or (5) home wound care. Community-acquired bacteremia was defined as patients with a positive blood culture obtained at the time of hospital admission or within 48 h after hospital admission and who did not fit the criteria for healthcare-associated infection [12, 13].

Patients with other sources of infection, history of intra-abdominal infection or recent abdominal surgery within the previous 3 m, as well as blood culture yielding anaerobes were excluded. Patients who did not receive appropriate definitive antibiotics therapy after the availability of their culture report, died before the antimicrobial susceptibility results were available, were transferred to another hospital before the completion of planned treatment course, or who were lost to follow-up after discharge were also excluded. In patients with multiple episodes of bacteremic BTI, only the first episode was included.

Antimicrobial regimens were selected by the clinicians according to the clinical condition of the patient and the guideline from Taiwan suggested anaerobic coverage in all kinds of intra-abdominal infections [14].

### Microbiological analysis

Blood samples were processed with BacT/Alert 3D (bioMérieux Inc., USA). All positive blood cultures set up for aerobic and anaerobic cultures. Species identification was done from mature colonies for all blood cultures. Bacterial identification from blood cultures was performed using matrix-assisted laser desorption-ionization time-of-flight mass spectrometry (bioMérieux, Marcy-l’Étoile, France). The minimal inhibitory concentration and antibiotic susceptibility were evaluated by the Vitek-2 system (bioMérieux), and the results were interpreted according to the 2014 CLSI criteria [15].

### Variables and definitions

The following information was collected: the patient demographics, underlying disease, biliary abnormalities, Pitt bacteremia score within 24 h before or on the day of positive blood culture, drainage procedure, appropriate empirical antibiotics, clinical response and total duration of antibiotic use. An empirical antibiotic was defined as any antibiotic that was first administered within 24 h after the blood samples were taken and continued for at least 48 h. Definitive antibiotics were defined as antibiotics that were continued or initiated on the day the susceptibility results were reported. Appropriate antimicrobial therapy was defined as treatment with at least one agent to which the isolate was susceptible in vitro according to the CLSI breakpoints [15]. Definitive therapy without anaerobic coverage was defined as cases in which anti-anaerobic agents were administered for less than 5 days and for less than 50% of the total duration of the therapy [9]. If these criteria were not met, the patients were classified into the group with anaerobic coverage.

All cases were followed-up until 14 days after discharge or to the time of death during the treatment course. The primary outcomes in this study were the clinical improvement, relapse, and 28-day mortality rates. Clinical improvement was defined as the resolution of all clinical signs and symptoms without recurrence or as successful discharge with oral antibiotics. Relapse was defined as the development of signs and symptoms similar to or worse than the index bacteremic BTI episode, within 14 days after discharge. Patients who died within 28 days whether relapse or not were categorized into the group with 28-day mortality. Treatment failure was defined as either relapse or 28-day mortality.

### Statistical analysis

The Chi-squared or Fisher’s exact test was used to analyze categorical data. The t-test was used to assess continuous variables. For all analyses, two-tailed tests were used to determine statistical significance and a value of *p* < 0.05 was considered significant. Univariate analysis was performed to evaluate the potential risk factors of treatment failure. Potential risk factors with *p* < 0.10 in the univariate analysis were included in the multivariate logistic regression model. Propensity score adjustment was used to indicate the probability of receiving anti-anaerobic treatment, modelled using logistic regression including age, sex, underlying disease (liver cirrhosis, diabetes mellitus, congestive heart failure, chronic kidney disease, chronic obstructive pulmonary diseases, and malignancy). and Pitt bacteremia score as the variables. To adjust for potential confounders, we included the propensity score data in the multivariate logistic regression to confirm our results. All statistical analyses were performed using SPSS version 22 (SPSS INC, Chicago, IL).

## Results

During the study period, a total of 103 patients with community-onset bacteremic BTIs were identified. Ten patients with incomplete medical records, five patients with hospital-onset BTIs, and one patient with anaerobic bacteremia were excluded. Finally, 87 patients were enrolled in this study. Among these patients, 61 patients were diagnosed with cholangitis, 21 patients with cholecystitis, and 5 patients with both cholecystitis and cholangitis.

Twenty-four patients received treatment without anaerobic coverage, while 63 received treatment with anaerobic coverage as the definitive therapy. Of the 87 patients, 65 (74.7%) were male, and the mean age of all patients was 73.4 ± 13.9 years. The most common biliary structure abnormality was choledocholithiasis (*n* = 52, 59.8%), followed by malignant obstruction (*n* = 31, 35.6%). The most common organism isolated from blood was *E. coli* (55.2%), followed by *K. pneumoniae* (23.0%). Seventy-three (83.9%) patients received biliary drainage and none of them had surgery during hospitalization. Only 12 patients received anaerobic bile cultures and anaerobes were isolated from 3 patients. The mean duration for obtaining antimicrobial susceptibility results of blood isolates was 2.93 days. The mean duration of antibiotic use was 13.18 days. The rates of clinical improvement, relapse, and 28-day mortality were 80.5, 14.9, and 4.6%, respectively.

The demographic and clinical characteristics of the patients are summarized in Table 1. We found no differences between the groups with and without anaerobic coverage as the definitive therapy in terms of their age, sex, biliary abnormalities, microbiology, and total duration of antibiotic use. The rate of appropriate empirical therapy between the groups with and without anaerobic coverage was similar (74.6% vs. 75.0%, *p* = 0.97). Patients in the group with anaerobic coverage as the definitive therapy also have a higher rate of empirical anaerobic coverage than those in the group without anaerobic coverage as the definitive therapy (88.9% vs. 50.0% *p* < 0.001). In the group without anaerobic coverage as the definitive therapy, the mean duration of empirical anaerobic coverage was 3.11 days. The disease severity as indicated by the Pitt bacteremia score, did not differ between the two groups. The outcomes of treatment with vs. without anaerobic coverage were similar in terms of the clinical improvement, relapse, and mortality rates. Three patients with anaerobes isolated from bile did not have treatment failure whether receiving anaerobic coverage (*n* = 1) or not (*n* = 2) (data not shown in Table 1).

**Table 1:** Characteristics and outcomes of patients with community-onset bacteremic BTIs with and without anaerobic coverage as the definitive therapy

Variables	Total (*N* = 87)	Definitive therapy without anaerobic coverage (*N* = 24)	Definitive therapy with anaerobic coverage (*N* = 63)	*p* value
Age (mean)	73.4 ± 13.9	71.5 ± 14.2	74.2 ± 13.9	0.419
Male sex	65(74.7)	18(75)	47 (74.6)	0.970
Underlying disease				
Diabetes mellitus	31(35.6)	8(33.3)	23(36.5)	0.782
Congestive heart failure	30(34.5)	3(12.5)	17(27.0)	0.151
Liver cirrhosis	4(4.6)	3(12.5)	1(1.6)	0.062
Chronic obstructive pulmonary diseases	10(11.5)	1(4.2)	9(14.3)	0.186
Chronic renal failure	68(78.2)	14(58.3)	54(85.7)	0.006
Malignancy	35(40.2)	9(37.5)	26(41.3)	0.749
Healthcare-associated bacteremia	38(43.7)	8(33.3)	30(47.6)	0.230
Microbiology				
*E.coli*	48(55.2)	16(66.7)	32(51.6)	0.207
*K. pneumoniae*	20(23.0)	2(8.7)	18(28.6)	0.053
Others^a^	28(32.2)	6(25.0)	22(34.9)	0.38
Biliary abnormalities				
Malignant obstruction	31(35.6)	7(29.3)	24(38.1)	0.437
Benign stricture	2(2.3)	0(0.0)	2(3.2)	1.000
Choledocholithiasis	52(59.8)	15(62.5)	37(58.7)	0.749
Unknown	2(2.3)	2(8.3)	0(0.0)	0.074
Pitt bacteremia score(mean)	1.2 ± 1.5	1.5 ± 1.8	1.1 ± 1.3	0.270
Biliary drainage^b^	73(83.9)	20(83.3)	53(84.1)	0.928
Appropriate empirical therapy	65(74.7)	18(75.0)	47(74.6)	0.970
Empirical therapy with anaerobic coverage	68(78.2)	12(50)	56(88.9)	< 0.001
Clinical outcome				
Clinical improvement	70(80.5)	20(83.3)	50(79.4)	0.677
Treatment failure	17(19.5)	4(16.7)	13(20.6)	0.677
Relapse	13(14.9)	3(12.5)	10(15.9)	0.693
28-day mortality	4(4.6)	1(4.2)	3(4.8)	1.000
Duration (days) of anaerobic coverage	9.1 ± 6.0	2.2 ± 1.8	11.8 ± 4.7	< 0.001
Total duration (days) of antibiotic use	13.2 ± 5.2	13.1 ± 5.7	13.2 ± 5.0	0.913

^a^Other pathogens included *Enterococcus* spp., *Streptococcus* spp., *Enterobacter* spp., *Pseudomonas* spp., *Raoultella* spp., *Acinetobacter* spp., *Citrobacter* spp., *Aeromonas* spp.

^b^Drainage included endoscopic retrograde cholangiopancreatography, percutaneous transhepatic cholangial drainage, percutaneous transhepatic gallbladder drainage, surgical drainage and indwelling stent

In this study, the treatment failure rate was 19.5%. The results of the univariate analysis of the associations between different variables and treatment failure are shown in Table 2. *K. pneumoniae* bacteremia, malignancy, healthcare-associated bacteremia, and higher Pitt bacteremia score were significant risk factors for treatment failure rates (*p* < 0.05). Variables with *p* values of < 0.1 and definitive therapy without anaerobic coverage were further analyzed in the multivariate analysis (Table 3). Malignancy (OR 11.27, 95% CI 1.78–71.67, *p* = 0.010) and higher Pitt bacteremia score (OR 2.27, 95% CI 1.36–3.80, *p* = 0.002) remained independent risk factors of treatment failure. Definitive therapy without anaerobic coverage did not predispose to treatment failure in this model (OR 2.67, 95% CI 0.34–21.31, *p* = 0.354). To adjust for possible confounders in the treatment groups, a propensity score adjustment was used in the multivariate analysis (Table 4). After adjustment, we further found *K. pneumoniae* bacteremia (OR 4.90, 95% CI 1.32–18.17, *p* = 0.018) and healthcare-associated bacteremia (OR 4.18, 95% CI 1.20–14.60, *p* = 0.025) to be related with the risk of treatment failure. Definitive treatment without anaerobic coverage was still not associated with an increased rate of treatment failure (OR 0.92, 95% CI 0.18–4.67, *p* = 0.916).

**Table 2:** Univariate analysis of the association between different variables and treatment failure in patients with community-onset bacteremic BTIs

Variables	OR	95%CI	*p* value
Age	1.02	0.98–1.06	0.384
Sex	1.84	0.59–5.76	0.294
Microbiology			
*E.coli*	0.48	0.16–1.41	0.180
*K. pneumoniae*	4.83	1.51–15.43	0.008
Other^a^	0.59	0.17-2.01	0.398
Underlying disease			
Liver cirrhosis	1.40	0.14–14.32	0.749
Diabetes mellitus	0.71	0.22–2.23	0.552
Congestive heart failure	1.04	0.30–3.63	0.953
Chronic obstructive pulmonary diseases	1.03	0.20–5.38	0.969
Chronic renal failure	5.54	0.69–44.79	0.108
Malignancy	4.90	1.54–15.59	0.007
Healthcare-associated bacteremia	4.06	1.29–12.83	0.017
Pitt bacteremia score	1.74	1.22–2.48	0.002
Appropriate empirical therapy	0.54	0.174–1.70	0.294
Definitive therapy without anti-anaerobic therapy	1.3	0.38–4.47	0.677
No drainage^b^	0.54	0.15–2.0	0.357

^a^Other pathogens included *Enterococcus* spp., *Streptococcus* spp., *Enterobacter* spp., *Pseudomonas* spp., *Raoultella* spp., *Acinetobacter* spp., *Citrobacter* spp., *Aeromonas* spp.

^b^Drainage included endoscopic retrograde cholangiopancreatography, percutaneous transhepatic cholangial drainage, percutaneous transhepatic gallbladder drainage, surgical drainage and indwelling stent

**Table 3:** Multivariate analysis of risk factors associated with treatment failure

Variables	OR	95%CI	*p* value
Age	1.03	0.97–1.09	0.355
Sex	0.73	0.14–3.79	0.711
*K. pneumoniae* bacteremia	3.63	0.81–16.23	0.091
Malignancy	11.27	1.78–71.67	0.010
Healthcare-associated bacteremia	2.17	0.49–9.65	0.311
Pitt bacteremia score	2.27	1.36–3.80	0.002
Definitive therapy without anti-anaerobic therapy	2.67	0.34–21.31	0.354

**Table 4:** Multivariate analysis of risk factors associated with treatment failure adjusted by propensity score^a^

Variables	OR	95%CI	*p* value
Age	1.01	0.96–1.06	0.730
Sex	1.27	0.33–4.90	0.724
*K. pneumoniae* bacteremia	4.90	1.32–18.17	0.018
Healthcare-associated bacteremia	4.18	1.20–14.60	0.025
Definitive therapy without anti-anaerobic therapy	0.92	0.18–4.67	0.916
Propensity score^a^	1.60	0.03–98.79	0.822

^a^Propensity score is the predicted probability of receiving treatment with anaerobic coverage, modelled by logistic regression with variables including age, sex, underlying disease (liver cirrhosis, diabetes mellitus, congestive heart failure, chronic renal failure, chronic obstructive pulmonary diseases, and malignancy), and Pitt bacteremia score

## Discussion

In the present study, we found that anaerobic coverage as definitive therapy was common (72.4%) in patients with community-onset bacteremic BTIs without anaerobes isolated from blood. However, we demonstrated that definitive therapy without anaerobic coverage did not affect the clinical outcomes among these patients.

In our study, we found only one anaerobic bacteremia, which is consistent to the low rate of anaerobes isolated from blood in the literature [2–6]. It has been reported that patients with clinical characteristics associated with biliary surgeries involving biliary-intestinal anastomoses and common bile duct manipulation are at higher risk for anaerobic infection [6, 7]. Accordingly, the Infectious Diseases Society of America (IDSA) guideline suggests empirical therapy with anaerobic coverage in patients with severe physiologic disturbance, advanced age, immunocompromised status, and acute cholangitis following bilio-enteric anastomosis, as well as in patients with healthcare-associated biliary infection [8]. However, it remains unclear whether anaerobic coverage is necessary after only aerobes have been isolated from the blood cultures, especially in patients without the above risk factors for anaerobic infections.

One randomized controlled trial conducted more than 20 years ago compared ciprofloxacin with triple therapy comprising ceftazidime, ampicillin, and metronidazole for the treatment of acute suppurative cholangitis. In the ciprofloxacin treatment arm, cases with anaerobic bacteria isolated from the blood cultures were excluded. The mortality, recurrence of fever, length of hospital stay, and need for emergent drainage were similar in the two treatment groups. These results indicate that antibiotics without anaerobic coverage are appropriate in patients with cholangitis but without anaerobic bacteria isolated from blood culture [16]. To the best of our knowledge, no other study addressing the issue about anaerobic coverage as the treatment for bacteremic BTIs has been reported to date.

One recent study, conducted by Kim et al.*,* reported that anaerobic coverage was not necessary for the treatment of *K. pneumoniae* liver abscesses [9]. This study enrolled patients who had liver abscesses with *K. pneumoniae* isolated from the blood or abscess and excluded those with polymicrobial or anaerobic infection. The authors compared the clinical outcomes between patients discontinuing and continuing the anti-anaerobic agents after *K. pneumoniae* were isolated, and found that the clinical outcomes for *K. pneumoniae* liver abscesses treated without anaerobic coverage was as good as those in patients treated with anaerobic coverage. In addition, these patients had a shorter length of hospital stay. These results provide some insight that anti-anaerobic agent might not be necessary in the treatment of other intra-abdominal infections if there is no evidence of anaerobic infection.

In this study, we selected patients with blood cultures positive for aerobes only and conducted propensity score analysis to adjust for the potential confounders among the treatment groups. It has been reported that acute renal failure, multiple underlying diseases, septic shock, the Pitt bacteremia score, and malignant obstruction are risk factors for mortality in bacteremic BTIs [2, 3]. In our study, we demonstrated that *K. pneumoniae* bacteremia and healthcare-associated infection were independent risk factors of treatment failure in the propensity-adjusted model. We also found that definitive therapy without anaerobic coverage does not affect the clinical outcomes. We hence suggest that anaerobic coverage as definitive therapy might not affect the clinical outcomes once the blood cultures come back negative for anaerobes.

The normal anaerobic gut flora plays an important role in the host defense by inhibiting the growth of potentially pathogenic microorganisms. Unnecessarily prolonged use of anaerobic agents leads to the growth of antibiotic-resistant microorganisms and increases the likelihood of vancomycin-resistant enterococcus colonization in the gastrointestinal tract and translocation into the bloodstream [17–20]. Narrowing down the antibiotic spectrum is crucial to prevent the selection of pathogenic organisms and the emergence of antibiotic resistance.

The IDSA guideline suggests 4–7 days of antibiotic treatment in complicated intra-abdominal infection [8]. The Tokyo guideline recommends similar treatment duration in BTIs once the source of infection is controlled [6]. However, treatment duration of bacteremic BTIs has not been well established. One recent study reported that nearly 70% patients with bacteremic cholangitis received 8 days or longer antimicrobial therapy even though they underwent successful biliary drainage [21]. Source control is important in the treatment of biliary tract infection, and more than 80% of patients received biliary drainage in the current study. Therefore, we suggested that the outcomes were similar between treatment with and without anti-anaerobic coverage in bacteremic BTIs in the current study based on adequate biliary drainage. This finding might give some insight on antibiotic strategy in treating patients with bacteremic BTIs.

The major limitation of our study is that it was a retrospective study, and there might hence exist some unmeasured variables and confounders in this study. However, we attempted to mitigate this by enrolling consecutive patients fitting the inclusion criteria and using propensity score analysis to adjust for the potential confounders. The limited case number was another limitation in this study, and caution must be taken in interpreting data from a small number of cases. Nevertheless, our study is, to our knowledge, the first to provide insight on this issue, and might inspire more studies regarding antimicrobial stewardship in this setting.

## Conclusions

Definitive therapy without anaerobic coverage does not affect the clinical outcomes of community-onset bacteremic BTIs without anaerobes isolated from blood. It implies that we could safely deescalate the antibiotics to cover aerobic bacteria only in cases where no anaerobes are isolated from the blood, provided that biliary drainage was performed. Our study might provide insights regarding possible targets for antibiotic stewardship interventions and provide a basis for further prospective studies.

## Abbreviations

BTI: Biliary tract infection
*E. coli*: *Escherichia coli*
IDSA: Infectious Diseases Society of America
*K. pneumoniae*: *Klebsiella pneumoniae*
